# Cellular Characterization and Neural Electrophysiology in Cerebral Organoid Models of Neurological Disorders

**DOI:** 10.14336/AD.2025.0758

**Published:** 2026-10-13

**Authors:** Quankun Zhou, Ling Ma, Bangming Guo, Peng Li, Ye Song

**Affiliations:** ^1^Department of Neurosurgery, Nanfang Hospital, Southern Medical University, Guangzhou, China.; ^2^Department of Neurosurgery, Guangzhou Women and Children's Medical Center, Guangzhou, China.; ^3^Department of Neurosurgery, The First Affiliated Hospital of Gannan Medical University, Ganzhou, China

**Keywords:** Cerebral organoids, Disease models, Cellular characteristics, Neuronal electrophysiology

## Abstract

Human cerebral organoids (hCOs), generated in vitro from induced pluripotent stem cells (iPSCs) or human embryonic stem cells (hESCs), exhibit cellular compositions similar to those of specific human brain regions. These organoids simulate early stages of brain development and can serve as in vitro disease models, providing a unique platform for studying neurodevelopmental processes and investigating underlying disease mechanisms. This review systematically summarizes research progress on cellular characteristics and neural electrophysiology in hCO-based models of neurodevelopmental disorders, neurodegenerative diseases, psychiatric disorders, epilepsy, viral infections, and traumatic brain injuries. These studies have elucidated the mechanisms underlying neuroelectrophysiological dysfunction in related diseases and facilitated innovative therapeutic explorations. Current limitations include prolonged culture durations and high costs, insufficient standardization that compromises reproducibility, the absence of neurovascular units that restrict pathological fidelity, immature laminar architectures that hinder complex circuit modeling, and electrophysiological bottlenecks in network-level analysis. Future efforts should focus on optimizing hCO culture protocols, innovating electrophysiological detection technologies, and promoting their clinical translation.

## 1. Introduction

In neuroscience research, investigations of the human brain are strictly constrained by ethical considerations, while traditional animal models struggle to accurately replicate the structural complexity and developmental specificity of the human brain due to interspecies differences [1, 2]. The breakthrough in human cerebral organoid (hCO) technology provides an innovative solution. hCOs, resembling the cellular composition and cytoarchitecture of the human brain, can be generated in vitro by inducing the differentiation of induced pluripotent stem cells (iPSCs) or human embryonic stem cells (hESCs) [3-5] (Table 1; Fig. 1). These models not only recapitulate early neurodevelopmental events but also replicate disease-specific pathological alterations (e.g., neurodegeneration, pathological protein aggregation) when derived from patient-derived iPSCs [6, 7].

Neural electrophysiology, as the functional output of neural networks, provides a dynamic observational window into the pathophysiological mechanisms in hCO-based disease models. For instance, Rett syndrome models with MECP2 mutations exhibit interneuron dysfunction and epileptiform discharge patterns [8], while Parkinson's disease models demonstrate dopaminergic neuron loss accompanied by abnormal oscillatory activity [9]. Schizophrenia models with *NRXN1* deletions reveal NMDA receptor-dependent deficits in network synchrony [10]. Notably, neuronal hyperexcitability in bipolar disorder models provides direct evidence for lithium's therapeutic mechanisms [11]. In contrast, Zika virus-induced selective destruction of neural progenitors and SARS-CoV-2-induced pathological hypersynchrony unveil distinct mechanisms underlying viral neurological complications [12, 13]. Furthermore, the combination of hCO transplantation and brain-computer interface technology in brain injury models demonstrates significant potential for restoring neural circuit function [14]. This review systematically synthesizes advances in cellular characterization and neural electrophysiology within hCO models of neurodevelopmental disorders, neurodegenerative diseases, psychiatric disorders, epilepsy, viral infections and traumatic brain injuries, aiming to establish a theoretical framework for mechanistic exploration and translational research.

**Table 1:** Culture Stages and Key Characteristics of Human Cerebral Organoids.

Stage	Time Frame	Experimental Procedures & Morphological/Structural Characteristics
Embryoid Body Formation	Days 0-5	hiPSCs undergo aggregation into embryoid bodies with subsequent proliferation.
Neural Induction	Days 6-10	Neural induction medium promotes development of translucent neuroectodermal tissue exhibiting pseudostratified epithelium at embryoid bodies periphery.
Neuroepithelial Budding	Days 11-15	Matrigel embedding combined with static culture enables emergence of polarized neuroepithelial buds displaying apical-basal polarity and ventricle-like lumina.
Brain Region Development	Days 15-30	Dynamic culture systems including spinning bioreactors or orbital shakers support differentiation into forebrain choroid plexus and hippocampal domains through dorsal-ventral patterning.
Long-term Maturation	Days 30-365	Progressive cortical layering occurs alongside regression of ventricular and subventricular zones with concurrent synaptogenesis.

## 2. Characteristics of Disease Models

### 2.1 Neurodevelopmental Disorders

#### 2.1.1 Primary Microcephaly

Primary microcephaly is associated with multiple genetic abnormalities. Its pathological mechanisms involve disruptions in key developmental processes, including the proliferation of neural stem/progenitor cells, cell cycle regulation, and neuronal differentiation/migration, ultimately leading to reduced head circumference and neurological dysfunction. Lancaster's team established an iPSC-derived hCO model of microcephaly, observing reduced neuronal soma size, diminished progenitor zones, decreased radial glial cell (RGC) populations, and premature neuronal differentiation. Exogenous introduction of *CDK5RAP2* rescued these phenotypes, confirming the central pathogenic role of its deficiency [4]. Further studies demonstrated that *NARS1* deletion in patient-derived cortical hCOs similarly reduced organoid volume, impairs RGC generation, and significantly decreased mature neuronal marker MAP2 expression, accompanied by disrupted neural rosette structures and morphological alterations in SOX2+ cells, indicating RGC proliferation failure leading to neuronal differentiation exhaustion [15]. PTEN overexpression models reveal that AKT pathway inhibition markedly delays hCO growth, expanding understanding of *PTEN*-related pathogenic mechanisms [16]. Unlike other microcephaly models that mainly show accelerated development or premature differentiation, the distinct phenotype in PTEN models may arise from its dual roles in neurodevelopment—regulating cell cycle exit via the AKT pathway while also modulating autophagy through the mTOR pathway. This mechanistic complexity highlights the risk of overinterpreting findings from single-gene models. Mitotic spindle marker TPX-2 and apical marker ZO-1 co-localization analyses demonstrate that the *AUTS2^T534P^* mutation doubles the frequency of asymmetric division in SOX2+ progenitors, suggesting this mutation disrupts normal neurodevelopmental trajectories by altering cell division modes [17]. Electrophysiological profiling of *ASPM* knockdown models shows that by day 85, mutant hCOs exhibit a 35% reduction in calcium-responsive cells and significantly reduced synchrony of spontaneous calcium transients compared to controls, confirming that progenitor proliferation defects cause delayed neural network maturation [18]. Both ASPM knockdown and NARS1 deletion lead to insufficient progenitor proliferation and reduced neuronal output, but their electrophysiological manifestations differ in timing and severity. This suggests that although different pathogenic genes converge on the same developmental pathway, they may still exert divergent functional consequences through gene-specific mechanisms. Recent studies applying low-intensity ultrasound to *ASPM*-mutant hCOs report increased cortical neuron numbers, elevated firing rates, and prolonged burst durations, demonstrating that physical stimulation can partially restore neural functionality [19]. Collectively, core electrophysiological hallmarks of microcephalic hCOs include attenuated electrical activity and reduced neural network synchrony, mechanistically linked to progenitor proliferation deficits and premature differentiation leading to insufficient mature neurons. Given the involvement of multiple genes in primary microcephaly, current research lacks sufficient mechanistic integration; therefore, the conclusions require validation through larger cohorts and multigenic mechanistic studies.

**Figure 1.**
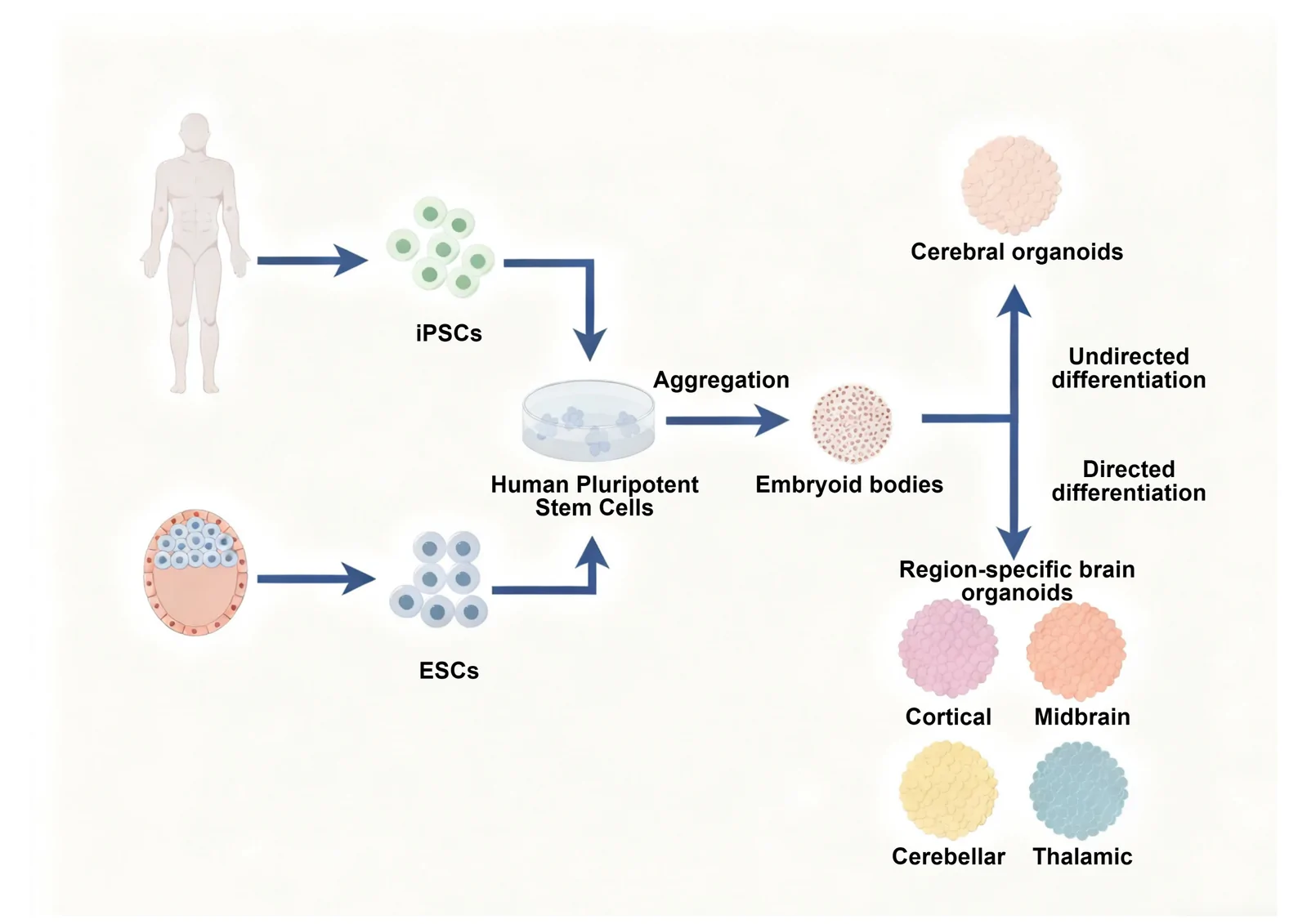
**Cultivation and differentiation process of human cerebral organoids (By Figdraw**. ID: IWIPU63346).

#### 2.1.2 Autism Spectrum Disorder

Autism Spectrum Disorder (ASD) is a childhood-onset neurodevelopmental condition characterized by deficits in social communication and repetitive sensorimotor behaviors. Its heritability ranges between 74-93%, involving heterogeneous genetic variants [20, 21]. To date, 102 ASD-risk genes have been identified, most of which participate in transcriptional regulation or neuronal communication during early brain development [22]. In genetic mutation models, single-cell sequencing of *SUV420H1*-, *ARID1B*-, and *CHD8*-mutant ASD-hCOs reveals premature GABAergic neuron differentiation, increased early-stage deep-layer projection neuron lineages in *SUV420H1* mutants, and asynchronous neuronal maturation across genetic backgrounds [23]. De novo heterozygous loss-of-function mutations in PTEN are strongly associated with ASD and result in pronounced developmental velocity disparities among specific cell types—including outer radial glia, deep-layer projection neurons, corticospinal neurons, and astrocytes—by month 3 [24]. Studies on hCOs derived from ASD patients without defined genetic abnormalities demonstrate: shortened neuronal cell cycles and elevated GABAergic neuron production; abnormal neuronal maturation, indicated by increased microtubule-associated protein 2 (MAP2) density; enhanced inhibitory synapse numbers; and upregulated gene expression in proliferation, neuronal differentiation, and synaptic assembly pathways [25]. Electrophysiological analyses show no statistical differences in voltage-gated sodium/potassium current amplitudes, action potential thresholds, or amplitudes between idiopathic ASD-hCOs and controls. However, accelerated sodium current inactivation at hyperpolarized membrane potentials is observed, potentially linked to increased Nav1.1 channel isoform expression [25]. In *SUV420H1*-mutant hCOs, neural network burst frequency and duration are reduced compared to controls, with greater NBQX-induced suppression of postsynaptic excitatory activity, confirming neuronal maturation disparities [23]. Conversely, *PTEN*-heterozygous mutant hCOs exhibit increased calcium oscillation burst frequency with shortened durations via calcium imaging, demonstrating opposing phenotypic effects of distinct risk genes on cortical circuit dynamics [24]. Collectively, these findings reveal multidimensional abnormalities in the timing of neuronal development, synaptic balance, and network activity in ASD patient-derived hCOs, thereby providing a robust model for mechanistic investigation. Notably, while some models (e.g., PTEN mutations) show enhanced neuronal activity, clinical ASD patients can display either increased or decreased brain electrical activity. This discrepancy likely reflects the inability of hCOs to capture region-specific circuit features. In addition, early network activity in hCOs does not clearly align with infant behavioral phenotypes, limiting their explanatory power. Future work should employ multi-regional assembloids, prolonged culture for maturation, and cross-species electrophysiological benchmarks to improve translational relevance.

#### 2.1.3 Attention-Deficit/Hyperactivity Disorder

Attention-Deficit/Hyperactivity Disorder (ADHD) typically manifests in childhood as developmentally inappropriate inattention, hyperactivity, and impulsivity, persistently impairing learning, social functioning, and daily living. Functional magnetic resonance imaging (fMRI) studies reveal functional abnormalities in attention- and executive-function-related neural networks during cognitive tasks in ADHD patients. Longitudinal studies have further demonstrated delayed cerebral maturation trajectories that progressively deviate from typical developmental patterns with age [26]. The risk of ADHD is associated with diverse genomic variants, with substantially elevated incidence observed in rare genetic syndromes, including fragile X syndrome and tuberous sclerosis complex (TSC) [26]. Patient-derived hCO models exhibit the following pathological features at day 56 of culture: significant reductions in neuroepithelial-like structures, cortical plate-like organization, and ventricular zone (VZ) thickness; decreased numbers of mitotic cells; increased apoptosis rates; and altered ratios of symmetric to asymmetric divisions. These findings collectively indicate early disruptions in neural progenitor proliferation and differentiation programs. Recent studies further support progenitor-level abnormalities in ADHD. Zhang et al. reported that telencephalon organoids derived from ADHD patients exhibited reduced cortical plate thickness and premature neuronal enrichment at days 35-56, followed by decreased proliferation and progressive VZ/CP thinning, suggesting a trajectory of "premature differentiation with proliferative exhaustion" [27]. Similarly, another study using ADHD patient-derived iPSCs revealed abnormal proliferative dynamics in vitro, reinforcing the notion that progenitor cell dysregulation may contribute to ADHD pathogenesis [28]. Notably, although existing ADHD organoid models recapitulate early proliferative and differentiation abnormalities, they do not replicate the circuit-level dysfunctions central to the disorder. While clinical studies emphasize alterations in dopaminergic and noradrenergic signaling, these systems remain inadequately modeled in hCOs. Moreover, early network activity patterns in organoids show no clear correlation with cognitive or behavioral phenotypes observed in patients. Most models focus on early developmental stages (35-56 days) and fail to capture later phases of network maturation, making it difficult to directly relate early proliferative-differentiation deficits to clinical impairments in attention and executive function. Furthermore, published findings are frequently derived from only one or a few patient-derived iPSC lines with limited genetic diversity and a lack of independent replication, which undermines the generalizability of the conclusions. Moving forward, research should prioritize long-term culture systems, multi-regional assembloids, and neurotransmitter-specific electrophysiological profiling to enhance the translational relevance of ADHD models.

#### 2.1.4 Rett Syndrome

Rett syndrome (RTT) is a severe, progressive neurodevelopmental disorder that predominantly affects females and is caused by mutations in the methyl-CpG-binding protein 2 (*MECP2*) gene. *MECP2* deficiency induces neuronal morphological abnormalities, including reduced soma size, shortened dendritic length, decreased dendritic complexity, and a reduced number of excitatory synapses. Electrophysiological studies demonstrate attenuated calcium transient activity—characterized by a lower proportion of active neurons and reduced oscillation frequency—in MECP2-deficient neurons, along with significantly decreased frequency and amplitude of spontaneous postsynaptic currents (sPSCs) [29-31]. Studies using hCO models reveal that MECP2 knockout reduces the numbers of outer radial glial cells (oRGs) and TUJ1+ neurons, depletes neural progenitor cells (hNPs) in the subventricular zone, increases the number of TUNEL+ apoptotic cells, and elevates the proportion of immature neurons, indicating impaired neurogenesis and neuronal maturation [32]. Whole-cell patch-clamp recordings of *MECP2e1*-mutant hCOs demonstrate elevated neuronal input resistance, reduced cell capacitance (associated with decreased somatic membrane area), significantly reduced voltage-gated sodium current amplitude and density, fewer evoked action potentials with diminished amplitude and prolonged duration, and decreased frequency and amplitude of miniature excitatory postsynaptic currents (mEPSCs), confirming synaptic connectivity deficits and neuronal immaturity [8]. Long-term cultured *MECP2* knockout (KO) hCOs exhibit further reductions in sodium and potassium current amplitudes and diminished mEPSC activity, suggesting a correlation between the severity of gene deletion and electrophysiological abnormalities [32]. Approximately 90% of individuals with RTT develop epilepsy during their lifetime, with mechanisms closely linked to interneuron dysfunction [33, 34]. A study analyzing epileptiform electrophysiological features in patient-derived cortical-ganglionic eminence (GE) fused hCO models reports increased synchronized calcium transients, reduced microcircuit cluster size, spike-like discharges, and high-frequency oscillations detected by local field potential (LFP) recordings, as well as decreased low-frequency and gamma-band oscillatory power [35]. Furthermore, comparative fusion strategies between control/mutant groups and cortical/GE hCOs confirm that *MECP2* deficiency in GE-derived interneurons serves as the core driver of neural network dysfunction in assembloids [35]. *MECP2*-mutant hCO models elucidate its impact on neurodevelopment and circuit stability, particularly regarding interneuron maturation, electrophysiological synchrony, and burst patterns. However, most current studies utilize complete knockout (KO) or e1-domain mutation models, which often produce severe electrophysiological abnormalities—such as reduced sodium current amplitude and decreased mEPSC frequency. In contrast, the majority of clinical RTT patients carry hypomorphic mutations (partial loss-of-function) rather than complete loss-of-function. Consequently, these models may overestimate the severity of electrophysiological impairments, thereby limiting their predictive value for evaluating gene therapies or pharmacologic interventions. The establishment of lineage-specific models incorporating various mutation types—such as common missense mutations, including *R106W*, *R133C*, and *T158M*—would more accurately recapitulate the clinical mutational spectrum. Although multiple studies consistently report attenuated calcium transient activity and reduced sPSC/mEPSC frequency, discrepancies exist regarding changes in specific currents (e.g., potassium currents) and action potential parameters. For instance, some studies report decreased potassium current amplitude, whereas others show no significant change. These inconsistencies may arise from: (1) the use of different mutation types (e.g., KO vs. point mutations) and genetic backgrounds of cell lines; (2) variations in organoid maturity (culture duration) and subtle differences in culture medium composition; (3) non-uniform electrophysiological recording parameters such as membrane potential holding levels and internal pipette solution formulations; and (4) intrinsic differences among neurons recorded from different regions of organoids (core vs. periphery).

#### 2.1.5 WOREE Syndrome

WOREE syndrome is a severe genetic disorder characterized by early-onset epileptic seizures in infancy or childhood, along with developmental delay, intellectual disability, and other neurological symptoms. It is strongly associated with germline pathogenic variants in the WWOX gene, and individuals carrying null alleles exhibit the most severe clinical manifestations [36]. Studies have shown that *WWOX* is specifically expressed in the VZ of cerebral organoids. Its knockout leads to upregulation of astrocytic markers (GFAP, S100β) and GABAergic neuronal markers, while glutamatergic neuronal marker expression remains unchanged [37]. In *WWOX* KO oligocortical hCOs at 14 weeks, mature oligodendrocytes (CC1+) are reduced without significant changes in oligodendrocyte precursor cells (PDGFRα+). Prolonged culture (20-30 weeks) leads to a marked reduction in myelination markers, including CNP and MBP. By week 37, electron microscopy reveals abundant unmyelinated axons, indicating severe hypomyelination [38]. Electrophysiological analyses demonstrate elevated action potential firing frequency and overall neuronal hyperactivity in patient-derived hCOs, reflecting neuronal hyperexcitability [37]. LFP monitoring in 7-week *WWOX* KO cortical hCOs exhibits significantly increased slow-wave oscillation (SWO, 0.25-1 Hz) power spectral density under baseline conditions. Upon induction with 4-aminopyridine (4-AP), electrical activity is further intensified, showing enhanced coupling between low-frequency and high-frequency oscillations and displaying typical epileptiform features [37]. Whole-cell patch-clamp recordings of 15-week *WWOX* KO oligocortical hCOs reveal depolarized resting membrane potentials (RMPs) closer to zero potential compared to wild-type and significantly enhanced SWO band (0.5-7.9 Hz) activity, consistent with seizure-like discharge phenotypes [38]. *WWOX*-mutant hCO models effectively reveal potential links among gliogenic aberrations, hypomyelination, and network hyperexcitability. However, current studies predominantly utilize complete WWOX knockout models, which limits their broader applicability. Furthermore, the hypothesis that myelination deficits directly contribute to enhanced network bursting is currently supported only by correlative evidence and lacks experimental validation of causality. It is important to note that existing research relies exclusively on complete knockout (KO) models, which differ significantly from the diverse hypomorphic mutations (partial loss-of-function) observed in clinical patients. Although null alleles lead to the most severe phenotypes, they represent only one part of the clinical mutational spectrum. This modeling extremity may exaggerate the severity of electrophysiological abnormalities (such as severely depolarized resting membrane potentials), thereby limiting their general predictive value for evaluating gene therapy strategies or pharmacological interventions. Although studies concurrently observe reduced myelination and network hyperactivity, only a temporal correlation exists between the two, lacking functional causal evidence. A central unanswered question is whether hypomyelination is the direct cause of enhanced network synchronization and epileptiform discharges, or whether both are independent parallel phenotypes resulting from WWOX deficiency. Causal direction needs to be validated through experiments that specifically promote myelination (e.g., using pro-myelinating small molecules) or inhibit neuronal activity followed by observing whether myelination improves.

#### 2.1.6 *PMM2* Deficiency

Mutations in the *PMM2* gene impair the catalytic activity of phosphomannomutase (PMM2), leading to abnormal protein N-glycosylation and subsequent multisystem dysfunction. Neurological manifestations of this defect include abnormal neuronal development, cerebellar ataxia, and epilepsy [39]. Studies using patient-derived hCOs confirm a significant positive correlation between PMM2 enzymatic activity levels and clinical central nervous system (CNS) severity scores [40]. Notably, although *PMM2*-mutant hCOs show no statistically significant differences in organoid diameter, neural stem cell marker expression, or spatial distribution patterns, transcriptomic analyses reveal significant downregulation of genes related to neurogenesis regulators (e.g., EOMES), synaptic plasticity-associated receptors (e.g., GRIN2B), and cortical development markers (e.g., TBR1, DCX), along with abnormal upregulation of cell adhesion molecules (PCDH family). These findings suggest that glycosylation defects may interfere with neuro-developmental processes through multiple molecular pathways [40]. At the electrophysiological level, multi-electrode array (MEA) recordings of patient-derived iPSC-generated neurons (iNeurons) show that PMM2-mutant cells maintain baseline spontaneous activity—including action potential firing frequency and network burst rates—comparable to controls, but exhibit significantly increased mean spike amplitudes and abnormal high-frequency oscillatory network patterns [40]. Although both transcriptomic abnormalities (e.g., downregulation of *GRIN2B*) and electrophysiological phenotypes (e.g., increased spike amplitude) have been observed concurrently, the molecular causal relationship between them remains unestablished. How glycosylation defects specifically lead to functional alterations in ion channels or receptors—such as impaired NMDA receptor function—and subsequently contribute to abnormal network activity still requires experimental validation. These electrophysiological abnormalities indicate that *PMM2* deficiency impairs the regulation of neural network rhythmicity. However, observations regarding high-frequency oscillations and firing rate alterations are currently limited to two-dimensional iNeuron networks derived from small sample sizes. These data remain preliminary in nature, and crucially, validation at the hCO level is lacking.

#### 2.1.7 Timothy Syndrome

Timothy syndrome (TS), caused by point mutations in the *CACNA1C* gene, induces functional abnormalities in L-type voltage-gated calcium channels (LTCs). This genetic defect disrupts critical neurodevelopmental processes including neuronal dendritic morphogenesis and cortical neuron migration, leading to neurological and psychiatric disorders such as epilepsy, autism spectrum disorder, ataxia, and schizophrenia [41, 42]. Studies using forebrain assembloid models generated from patient-derived human cortical spheroids (hCSs) and human subthalamic spheroids (hSSs) reveal significant interneuron migration defects in TS, manifested as reduced migration distance and increased migration frequency (indicating decreased efficiency), with impaired rear contraction dynamics during migration causing spatial decoupling from the leading edge (soma polarity dysregulation) [42]. In transplantation models, TS patient-derived hCOs grafted into the primary somatosensory cortex (S1) of neonatal athymic rats show 200% increased primary dendrite number compared to controls, alongside reduced average/total dendritic lengths and elevated synaptic spine density [43]. Electrophysiologically, calcium imaging demonstrates significantly increased frequency of spontaneous calcium transients in TS-hSS neurons. Patch-clamp recordings reveal hyperpolarized RMPs and increased GABA-evoked current amplitudes in interneurons, suggesting heightened intrinsic excitability and GABAergic responsiveness in TS-hSS neurons [42]. Another study identifies multiple electrophysiological abnormalities in TS-hCO neurons: elevated membrane capacitance, increased action potential amplitudes, enhanced maximum firing rates, and heightened frequency of spontaneous excitatory postsynaptic currents (sEPSCs) [43]. Molecular analyses confirm delayed inactivation kinetics of CaV1.2 channels and aberrant exon 8A splicing in TS-hCOs. Antisense oligonucleotides (ASOs) targeting *CACNA1C* effectively correct calcium channel splicing defects, restore calcium channel function in patient neurons, and improve interneuron migration efficiency, thereby ameliorating TS-associated neurobiological abnormalities [44]. However, this study did not assess ASO effects on electrophysiological phenotypes. Establishing hCO-based electrophysiological pharmacodynamic evaluation systems will help validate correlations between channel functional rescue and neural network activity normalization, quantify therapeutic modulation of neuronal excitability imbalances, and provide critical evidence for clinical translation. However, current research predominantly focuses on early developmental stages, and patient-derived hCOs model a single mutation site (G406R). Their capacity to recapitulate other mutational subtypes or distinct clinical variants remains unestablished. While ASO interventions demonstrate correction of splice-site abnormalities, whether they restore electrophysiological functions and network synchrony lacks supporting data. The existing models focus exclusively on a single mutation site (G406R) and do not encompass other known pathogenic CACNA1C mutations (e.g., G402S), limiting their ability to account for genotype-phenotype heterogeneity in TS. Although ASO interventions can correct splicing defects and migration phenotypes, there is a lack of validation regarding their effects on electrophysiological functions (such as increased sEPSC frequency and abnormal action potential parameters) and network synchrony, making it difficult to establish a causal link between molecular repair and functional improvement. Current studies primarily address early developmental events (e.g., neuronal migration) but lack longitudinal tracking of how these mutations impact later network maturation, such as the emergence of epileptiform activity.

#### 2.1.8 *UBA5*-Related Encephalopathy

Pathogenic variants in the *UBA5* gene are strongly associated with neurodevelopmental disorders and epileptic encephalopathy, clinically manifested as severe developmental delay, hypotonia, spastic paralysis, microcephaly, and intractable epilepsy. Studies demonstrate that patient-derived cortical hCOs exhibit a 25% reduction in volume compared to healthy controls, successfully recapitulating the pathological morphological features of clinical microcephaly. Mechanistic analyses reveal significantly decreased expression levels of rate-limiting enzymes for GABA synthesis (GAD1/GAD2), leading to impaired differentiation and reduced populations of GABAergic neurons. These developmental defects in inhibitory circuitry likely disrupt cortical excitation/inhibition (E/I) balance, forming the pathological basis of epileptiform network abnormalities. MEA recordings show that *UBA5*-mutant hCOs display significantly elevated weighted mean firing rates and average burst frequencies compared to controls, indicating enhanced spontaneous neuronal activity. Concurrently, reduced synchronized network burst frequencies and shortened burst durations suggest impaired temporal coordination of neural network activity [45]. However, it remains unclear why UBA5 deficiency selectively affects the GABAergic lineage rather than all neuronal populations, and the mechanism underlying this specificity has yet to be elucidated. It is also uncertain whether the downregulation of GAD expression is the direct cause of E/I imbalance or a secondary effect resulting from broadly impaired cellular metabolism. Functional rescue experiments, such as GAD overexpression, are required to validate this causality. Moreover, current models are based on severe loss-of-function mutations, and it remains unclear whether they can recapitulate the milder phenotypes observed in patients with partial loss-of-function mutations.

Multiple disease models (e.g., microcephaly, Rett syndrome, UBA5-related encephalopathy) consistently demonstrate impaired neural progenitor proliferation or disrupted differentiation, ultimately leading to reduced mature neuron populations and attenuated network electrophysiological activity. Furthermore, diminished network synchrony and aberrant burst patterns are commonly observed across epilepsy-associated models. In contrast, ASD and TS models predominantly manifest neuronal migration defects, synaptic equilibrium abnormalities, and elevated firing frequencies—features indicative of "developmental-functional decoupling." Meanwhile, WOREE models reveal connections between disturbances in gliogenesis and neural network dysrhythmia, whereas PMM2 deficiency models suggest that glycosylation defects may disrupt neuronal maturation and network rhythmicity through multiple pathways. However, these studies remain predominantly based on iPSCs from individual patients or specific gene-edited cells, constrained by restricted sample sizes and homogeneous genetic backgrounds (Fig. 2).

### 2.2 Neurodegenerative Diseases

#### 2.2.1 Parkinson's Disease

Parkinson's disease (PD) is pathologically characterized by progressive degeneration of midbrain substantia nigra-striatal dopaminergic neurons and α-synuclein pathology (Lewy bodies and neurites). Its clinical triad includes bradykinesia, resting tremor, and rigidity, often accompanied by non-motor symptoms such as autonomic dysfunction and cognitive impairment [46]. Genetic studies confirm associations between PD pathogenesis and variants in *LRRK2*, *DNAJC6*, and *PINK1* [7, 9, 47, 48]. Among these, *LRRK2* mutations play significant roles in both familial and sporadic PD [47], though hCOs carrying the *LRRK2^G2019S^* mutation fail to reproduce classical Lewy body pathology [9]. In contrast, hCO models of *DNAJC6* and biallelic *PINK1* mutations successfully replicate abnormal α-synuclein monomer/ oligomer accumulation [7, 48]. Mechanistically, *DNAJC6* deficiency induces mitochondrial dysfunction and autophagosome stasis in hCO neurons [7], while *PINK1* mutations impair differentiation of neuroepithelial stem cells (NESCs) into dopaminergic neurons (TH+), concurrent with NESC hyperproliferation (elevated Ki67+ ratios), TH+ neuronal apoptosis (enhanced cPARP signaling), and astroglial activation (increased GFAP+/ S100β+ cells) [48]. Electrophysiological analyses reveal that *LRRK2^G2019S^* mutant hCOs show no neuronal activity differences from controls during early culture (<6 months). However, prolonged culture (6-10 months) demonstrates progressive neural network dysfunction: reduced network burst rates, prolonged network event periods, and weakened spike correlation-based connectivity. Spectral analyses identify abnormal power increases in delta-band (<4 Hz), theta-band (5-8 Hz), and beta-band (14-32 Hz) oscillations, alongside significant reductions in low gamma-band (33-80 Hz) power and disrupted high gamma (100-200 Hz) synchrony, suggesting E/I circuit imbalance [9]. Notably, *DNAJC6*-mutant hCOs exhibit a twofold elevation in neuronal firing frequency at day 80 via MEA recordings, mirroring the "stressful pacing" electrophysiological signatures observed in PD patients [7]. These findings systematically elucidate genetic subtype-specific neuroelectro-physiological features and molecular mechanisms, providing critical experimental insights for deciphering PD pathological heterogeneity and developing targeted therapies. However, current *LRRK2^G2019S^* hCOs consistently fail to form Lewy body-like inclusions, whereas DNAJC6 and PINK1 mutants readily accumulate α-synuclein aggregates, suggesting that Lewy body pathology may require additional extrinsic stressors or glial contributions absent in current organoids. Electrophysiological alterations are mutation- and model-dependent: DNAJC6 mutants exhibit early hyperexcitability, while LRRK2 mutants show delayed network hypoactivity and spectral shifts after prolonged culture, indicating distinct temporal trajectories among PD subtypes. Common features such as mitochondrial dysfunction, autophagy deficits, and dopaminergic neuron loss are observed across mutations, but whether these represent convergent PD hallmarks or mutation-specific effects remain unclear. Moreover, current models largely focus on rare familial mutations, leaving sporadic PD—representing most cases—understudied, with minimal incorporation of environmental risk factors, aging effects, or gene-environment interactions.

**Figure 2.**
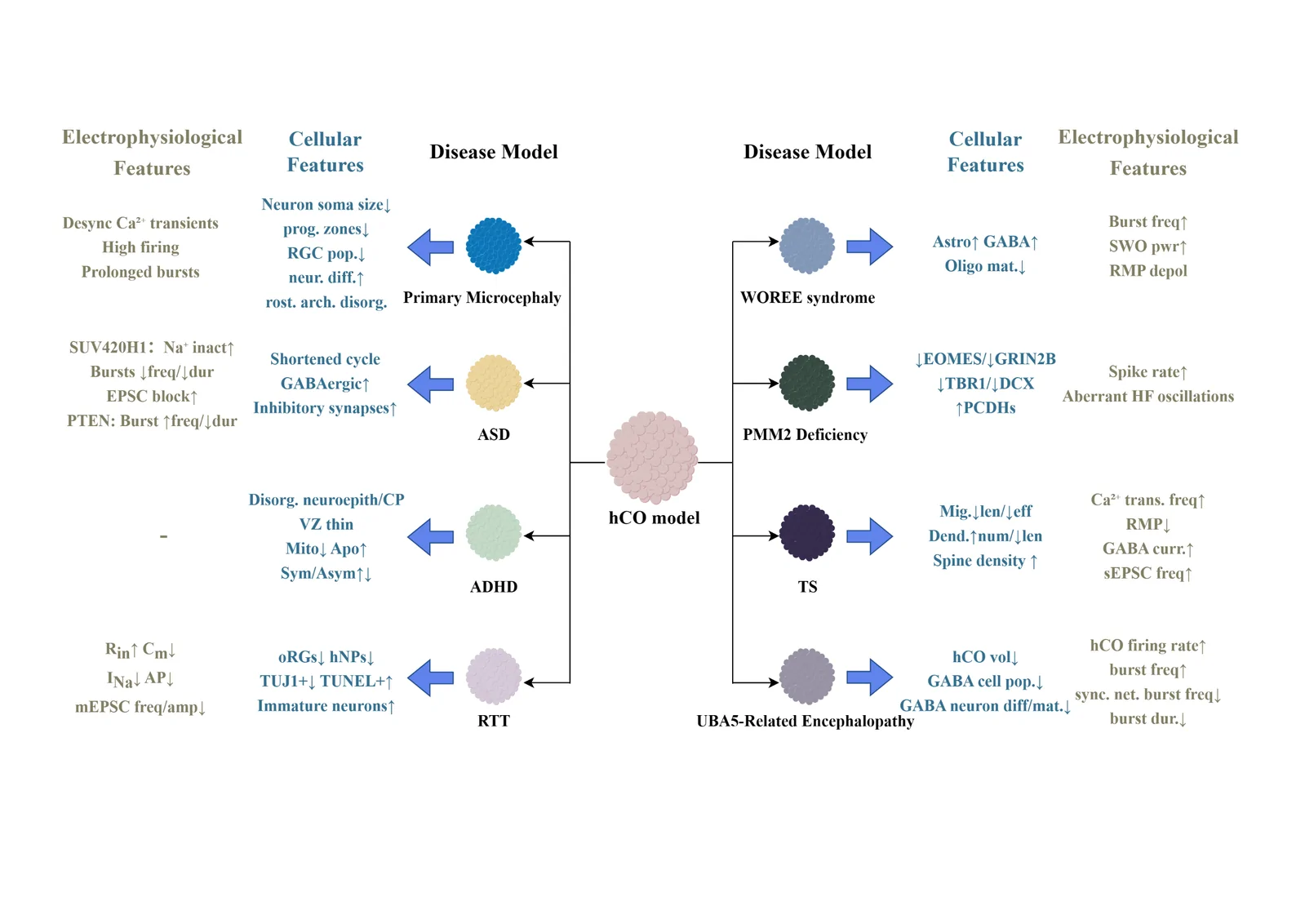
**Cellular and electrophysiological features of hCO models for neurodevelopmental disorders (By Figdraw**. ID: TIYRWe1f6a).

#### 2.2.2 Alzheimer's Disease

Alzheimer's disease (AD) is an irreversible progressive neurodegenerative disorder characterized by dual pathological hallmarks: extracellular β-amyloid (Aβ) plaque deposition and intraneuronal phosphorylated tau (p-tau) neurofibrillary tangles. Familial AD (fAD) is predominantly driven by mutations in the amyloid precursor protein (APP) and presenilin (PSEN1/PSEN2) genes, while apolipoprotein E4 (*APOE4*) serves as a major genetic risk factor for sporadic AD (sAD) [49, 50]. CRISPR/Cas9-engineered isogenic *APOE* models demonstrate that *APOE3*-hCOs exhibit significantly higher astrocytic APOE protein expression and enhanced Aβ phagocytic clearance compared to *APOE4*-hCOs. Conversely, *APOE4*-hCOs display pathological Aβ accumulation and p-tau aggregation, confirming that *APOE4* exacerbates sAD pathology by impairing glial functions [6]. Chimeric hCO studies further reveal that co-expression of *APOE4* in both astrocytes and neurons is required to elevate neuronal p-tau levels, highlighting the critical role of astrocytes in AD pathogenesis [51]. Electrophysiological investigations show increased mEPSC frequencies in *APOE4*-carrier iPSC-derived neurons compared to *APOE3* controls, indicating synaptic plasticity abnormalities [6]. Blood-brain barrier (BBB) leakage represents another AD risk factor. Serum-exposed hCO models mimicking BBB leakage demonstrate that exogenous serum upregulates BACE expression and enhances GSK3 activity, promoting Aβ deposition and p-tau accumulation. Concurrently, synaptic marker SYN1 expression decreases alongside suppressed neural network activity (reduced calcium signals, diminished spikes/burst counts, lower mean firing rates, and decreased synchronization indices) [52]. Currently, AD-specific neuroelectrophysiological signatures in hCO models remain underexplored, warranting further investigation. However, AD-hCO models predominantly rely on familial mutations (*APP/PSEN1*) or APOE4 backgrounds, limiting their capacity to simulate the insidious progression characteristic of most sporadic AD cases. In some studies, the correlation between diminished network activity and synaptic loss remains unestablished, while systematic validation between calcium imaging and MEA data is lacking. Furthermore, due to culture period constraints, current AD-related hCO models primarily represent early disease stages, and their ability to stably recapitulate late-stage tau pathology through extended culture requires further investigation. While hCO models have provided valuable insights into AD mechanisms, several critical limitations require attention. First, although APOE4 models consistently exhibit increased Aβ and p-tau accumulation with impaired glial clearance, reported electrophysiological outcomes remain conflicting. Some studies report elevated mEPSC frequency, implying synaptic hyperactivity, while serum-exposure or BBB impairment models demonstrate suppressed network activity and reduced synchronization. This inconsistency may reflect stage-dependent pathological transitions from early hyperexcitability to synaptic failure, but also highlights the absence of standardized electro-physiological protocols across studies. Second, commonly reported overlapping findings—including reduced network synchrony, synaptic marker loss, and Aβ/p-tau accumulation—have not been systematically integrated, leaving it uncertain whether they represent convergent disease hallmarks or methodological artifacts. Third, most existing models depend on familial mutations or APOE4 backgrounds, failing to capture the multifactorial etiology of sporadic AD. Environmental risk factors, aging-related changes, and vascular contributions such as chronic BBB dysfunction are largely unrepresented, diminishing clinical translatability. Finally, culture duration poses a persistent constraint; although tau aggregation has been observed, the robust reproduction of stable long-term and progressive late-stage AD pathology remains unrealized in vitro. These observations remain confined to early developmental stages, limiting their ability to model the long-term progression characteristic of sporadic AD.

#### 2.2.3 *C9ORF72* Hexanucleotide Repeat Expansion

The hexanucleotide repeat expansion (HRE) in the *C9ORF72* gene is a core genetic determinant of amyotrophic lateral sclerosis (ALS) and frontotemporal dementia (FTD). Its pathological mechanisms involve multiple pathways, including loss of C9ORF72 protein function, toxic RNA repeat accumulation, and aggregation of repeat-associated non-ATG (RAN)-translated dipeptide repeat proteins (DPRs) [53]. Studies using patient-specific cerebral organoids (C9 ALI-COs) demonstrate preserved cortical cell type diversity and synaptic density compared to controls [54], yet reveal characteristic dynamic alterations during development, including early-stage abnormal enlargement of organoid cross-sectional area, accompanied by progressive C9ORF72 downregulation and poly(GA)/poly(GP) DPR accumulation. Single-cell transcriptomic profiling further shows reduced deep-layer neuron (CTIP2+) populations at late stages (day 90), altered SOX2^+^ radial glial cell distribution and clustering, and abnormal increases in upper-layer neuron-to-glial ratios [55]. Three-dimensional multi-electrode array (3D-MEA) recordings of long-term cultured (150-193 days) C9 ALI-CO slices show no statistical differences in spontaneous activity parameters (spike frequency, time coverage fraction) versus controls [54]; however, patch-clamp studies detect significant resting membrane potential depolarization, reduced sEPSC frequency, and declining action potential success rates in C9 ALI-CO neurons [55]. Denervation-induced muscular atrophy, a hallmark of ALS-related muscle weakness, has been modeled using patient-derived neuromuscular organoids (NMOs). C9-HRE NMOs exhibit reduced contraction frequency by day 50 and significant decreases in neuromuscular junction (NMJ+) numbers, acetylcholine receptor cluster areas, and adjacent S100+ Schwann cell populations by day 100. These organoids also show upregulated autophagy markers (p62/LC3b) in neurons and astrocytes, alongside diminished calcium transient peak amplitudes via calcium imaging, despite unaltered spike amplitudes, frequencies, or durations in MEA recordings [56]. Long-term 3D-MEA recordings in C9 ALI-COs show no significant differences in spontaneous spiking compared to controls, whereas patch-clamp analyses reveal neuronal depolarization, reduced sEPSC frequency, and declining action potential fidelity. This highlights a discrepancy between population-level and single-cell measurements, suggesting that subtle biophysical defects may not immediately manifest at the network level or may be masked by compensatory mechanisms. Similarly, NMOs exhibit structural and functional NMJ deficits—reduced NMJ density, impaired AChR clustering, and diminished Schwann cell support—yet MEA activity remains relatively intact despite lower calcium transient amplitudes, emphasizing potential divergence between synaptic impairments and gross electrophysiological outputs. Recurrent observations of autophagy dysregulation, radial glial alterations, and DPR accumulation are noted, but it remains unclear whether these reflect convergent disease hallmarks or context-specific effects of C9-HRE. Current ALS-hCO models are largely limited to early developmental time frames, falling short of replicating the chronic progression of motor neuron loss observed in vivo. Finally, reliance on patient-derived iPSCs from limited pedigrees may introduce biases, reflecting individual genetic modifiers rather than universal mechanisms. Broader use of CRISPR-based isogenic C9-HRE models, longitudinal multi-modal electrophysiology, and integration with neuromuscular assembloids will be essential to resolve these inconsistencies and clarify how molecular pathology drives progressive motor circuit dysfunction. Neurodegenerative disease models collectively reveal associations between pathological protein accumulation and electrophysiological disturbances, albeit through divergent mechanisms: in Parkinson's disease, *LRRK2* mutations cause late-stage gamma oscillation attenuation, whereas *DNAJC6* mutations elicit early-phase firing frequency elevation. Similarly, synaptic plasticity aberrations (elevated mEPSC frequency) in *APOE4*-based AD models contrast with resting membrane potential depolarization in C9ORF72 models. Notably, all models exhibit temporally aggravated electrophysiological deterioration, yet are driven by distinct pathogenic cascades.

### 2.3 Psychiatric Disorders

#### 2.3.1 Major Depressive Disorder

The pathophysiological mechanisms of major depressive disorder (MDD) remain incompletely elucidated. Neuroimaging studies indicate its close association with neuronal connectivity remodeling and network dysfunction in cortical and limbic systems [57]. In patient-derived models of severe major depressive disorder with suicidal behavior (sMDD), studies utilizing iPSC-differentiated GABAergic interneurons (GINs) and ventral forebrain organoids have revealed the following findings: sMDD-derived GINs exhibit significantly increased neurite complexity, manifested as higher intersection counts, maximum intersection density, and total intersections within 10-, 30-, and 50-μm concentric radii from the soma compared to controls, alongside abnormal increases in primary branch numbers and maximal neurite lengths of mature GINs. Electrophysiological analyses via whole-cell patch-clamp recordings demonstrate reduced action potential firing thresholds, elevated action potential amplitudes and firing frequencies, and an approximately 56% reduction in action potential half-width compared to controls, accompanied by enhanced inward sodium current and rapid potassium current amplitudes, suggesting ion channel dysfunction as a potential molecular basis for excitatory-inhibitory imbalance. Notably, calcium imaging in sMDD ventral forebrain organoids shows significantly diminished neuronal calcium signal amplitudes relative to controls, indicating calcium homeostasis dysregulation [58]. Electrophysiological findings in sMDD organoids vary across platforms: patch-clamp recordings indicate increased excitability (lower firing thresholds, higher amplitudes, and frequencies), whereas calcium imaging shows reduced signal amplitudes, suggesting that ionic currents and intracellular calcium dynamics may follow distinct or opposing trajectories. The source of this discrepancy—methodological sensitivity, developmental stage, or compensatory mechanisms—remains unclear. Most data derive from GABAergic interneurons or ventral forebrain organoids from a small number of iPSC donors, limiting generalizability to broader MDD populations. Recurring features such as abnormal neurite complexity and altered ion channel function have been reported, but systematic replication is lacking. Moreover, cortical-limbic connectivity deficits, consistently observed in patient neuroimaging, are largely unmodeled in current organoid systems. Addressing these gaps will require larger, genetically and ethnically diverse cohorts, long-term cortical-limbic assembloid models, and multi-modal electrophysiology to directly link neuronal hyperactivity with calcium dysregulation.

#### 2.3.2 Bipolar Disorder

Bipolar disorder (BD) is clinically defined by recurrent episodes of extreme mood fluctuations accompanied by significant cognitive impairment [59]. Patient-derived cortical hCO studies reveal reduced average organoid size, downregulated cortical layer markers (CTIP2, SATB2, TBR1), and synaptic protein SYN1 expression [11], decreased neuroepithelial bud (NEB) counts with disorganized spatial arrangement, and diminished VZ area leading to impaired NEB formation due to reduced SOX2+ progenitor populations, suggesting early neurodevelopmental deficits [60]. Abnormal increases in neural rosette numbers with structural disorganization, loss of laminar architecture, and imbalanced mixed-cell ratios further indicate inherent neurodevelopmental vulnerability in BD-hCOs [61]. Transcriptomic analyses demonstrate downregulation of neurodevelopment-, neurogenesis-, synaptic signaling-, and neuronal projection-related genes, alongside upregulated mitochondrial respiratory chain genes in BD-hCOs [60-62]. Notably, under physiological oxygen levels (5%), BD-hCOs show reversed mitochondrial ribosomal gene downregulation and partial rescue of rosette abnormalities, confirming that supraphysiological oxygen exacerbates metabolic and neurodevelopmental defects [61]. Electrophysiological studies reveal unaltered baseline spontaneous firing rates in BD-hCOs via MEA recordings, but impaired depolarization-evoked firing responses. Patch-clamp analyses detect a rightward shift in whole-cell current-voltage (I-V) curves and elevated threshold voltages for maximum current intensity (Imax), indicating neuronal hypoexcitability [11, 62]. Paradoxically, calcium imaging demonstrates hyperactive network states in BD-hCOs with increased calcium transient peak amplitudes, shortened intervals, and enhanced synchrony [61]. Pharmacological investigations show lithium exerts differential effects: it significantly enhances neuronal excitability in BD-hCOs while suppressing controls. Although sEPSC frequencies increase across groups, BD-hCOs exhibit reduced sEPSC amplitudes post-lithium treatment [11]. Crucially, lithium fully restores abnormal neuronal synchrony in BD-hCOs, demonstrating its therapeutic potential to rebalance neural networks [61]. BD-hCO models reveal early neuro-developmental disruptions characterized by disordered cellular lamination, downregulated synaptic markers, and network functional imbalance. Patch-clamp studies consistently show neuronal hypoexcitability in BD-hCOs, yet calcium imaging paradoxically reveals hyperactive network states, raising questions about how single-cell dysfunction translates into network behavior. One possibility is that reduced excitability at the cellular level is compensated by abnormal synchronization among surviving networks, but without simultaneous patch-clamp and Ca²^+^ imaging, this remains unresolved. Transcriptomic analyses repeatedly show downregulation of neurodevelopmental and synaptic genes and upregulation of mitochondrial pathways, though it is unclear whether these are primary disease mechanisms or secondary stress responses to culture conditions. The finding that physiological oxygen levels can rescue some phenotypes highlights the influence of non-genetic environmental factors, complicating interpretation of disease-specific effects. Finally, lithium shows differential effects—restoring synchrony in BD-hCOs while suppressing excitability in controls—suggesting potential patient subtypes, yet current models rarely integrate clinical features or treatment response.

#### 2.3.3 Schizophrenia

Schizophrenia (SCZ) is clinically characterized by psychotic symptoms (e.g., hallucinations, delusions) and cognitive dysfunction, with well-established genetic susceptibility yet highly heterogeneous molecular mechanisms [63]. Patient-derived hCOs exhibit reduced VZ volume, decreased cortical neuron numbers, increased neuroprogenitor apoptosis, and abnormal differentiation bias (elevated non-neuronal lineage commitment) [64]. Specific genetic models, such as *NRXN1*-deficient hCOs, demonstrate aberrant neural progenitor differentiation trajectories as early as 3 weeks of development, suggesting synaptic adhesion molecule defects may disrupt neurodevelopmental programming [10]. Transcriptomic analyses reveal significant down-regulation of genes involved in nervous system development, neurogenesis, and neuronal differentiation, alongside aberrant activation of immune response and extracellular matrix regulation genes [65]. Electrophysiological studies uncover complex functional abnormalities in SCZ: MEA recordings show blunted depolarization-evoked firing frequency responses in patient-derived hCOs, indicating neuronal hypoexcitability [65]. Calcium imaging of *NRXN1*-deficient hCOs demonstrates markedly reduced frequency of spontaneous calcium transients and synchrony, with failure to enhance neuronal activity upon chemical long-term potentiation (cLTP) induction, confirming N-methyl-D-aspartate receptor (NMDAR) dysfunction may disrupt synaptic transmission and network synchronization. These electrophysiological phenotypes persist at later maturation stages [10]. Interestingly, gene-edited NRXN1 knockout models exhibit increased calcium transient frequency, highlighting that the impact of NRXN1 deletion on neuronal electrophysiological activity varies across genetic backgrounds [10]. *PCCB*-knockdown hCOs display neuronal hyperexcitability and reduced network synchrony, mirroring electro-encephalographic (EEG) signatures observed in SCZ patients [66]. Thalamocortical assembloids with *CACNA1G* deficiency show elevated spontaneous firing frequencies in thalamic neurons, increased thalamocortical axonal projections, and diminished interneuronal synchrony, implicating voltage-gated calcium channel defects in thalamocortical circuit dysfunction during SCZ pathogenesis [67]. A key inconsistency in NRXN1 studies is that patient-derived hCOs show reduced neuronal activity and impaired Ca²^+^ synchronization, whereas CRISPR-edited NRXN1 knockouts display increased calcium transient frequency, suggesting that genetic background and polygenic modifiers strongly influence electrophysiological outcomes. Similarly, PCCB-knockdown and CACNA1G-deficient models both exhibit hyperexcitability but differ at the network level—reduced synchrony in PCCB organoids versus thalamocortical hyperconnectivity in CACNA1G assembloids—raising the question of whether these reflect shared E/I imbalances or mutation-specific mechanisms. Overlapping transcriptomic patterns, such as downregulation of neurogenesis genes and activation of immune pathways, recur across studies, but it is unclear whether these are SCZ-specific hallmarks or general culture stress responses. Finally, most SCZ-hCO models are limited to early developmental stages, restricting their ability to model chronic disease trajectories and higher-order cognitive dysfunctions.

While both BD and MDD models demonstrate neuronal morphological alterations, their electrophysiological profiles diverge: BD models display enhanced calcium signal synchrony, whereas sMDD models primarily manifest ion channel hyperfunction. The NRXN1-deficient SCZ model further reveals bidirectional modulation of electrical activity by genetic context—patient-derived models exhibit neural activity suppression, whereas CRISPR-edited counterparts show hyperactivity—indicating that identical mutations can produce diametrically opposed electrophysiological effects depending on genetic background. Notably, lithium's specific correction of synchrony in BD models provides experimental support for subtype-specific therapeutics.

**Figure 3.**
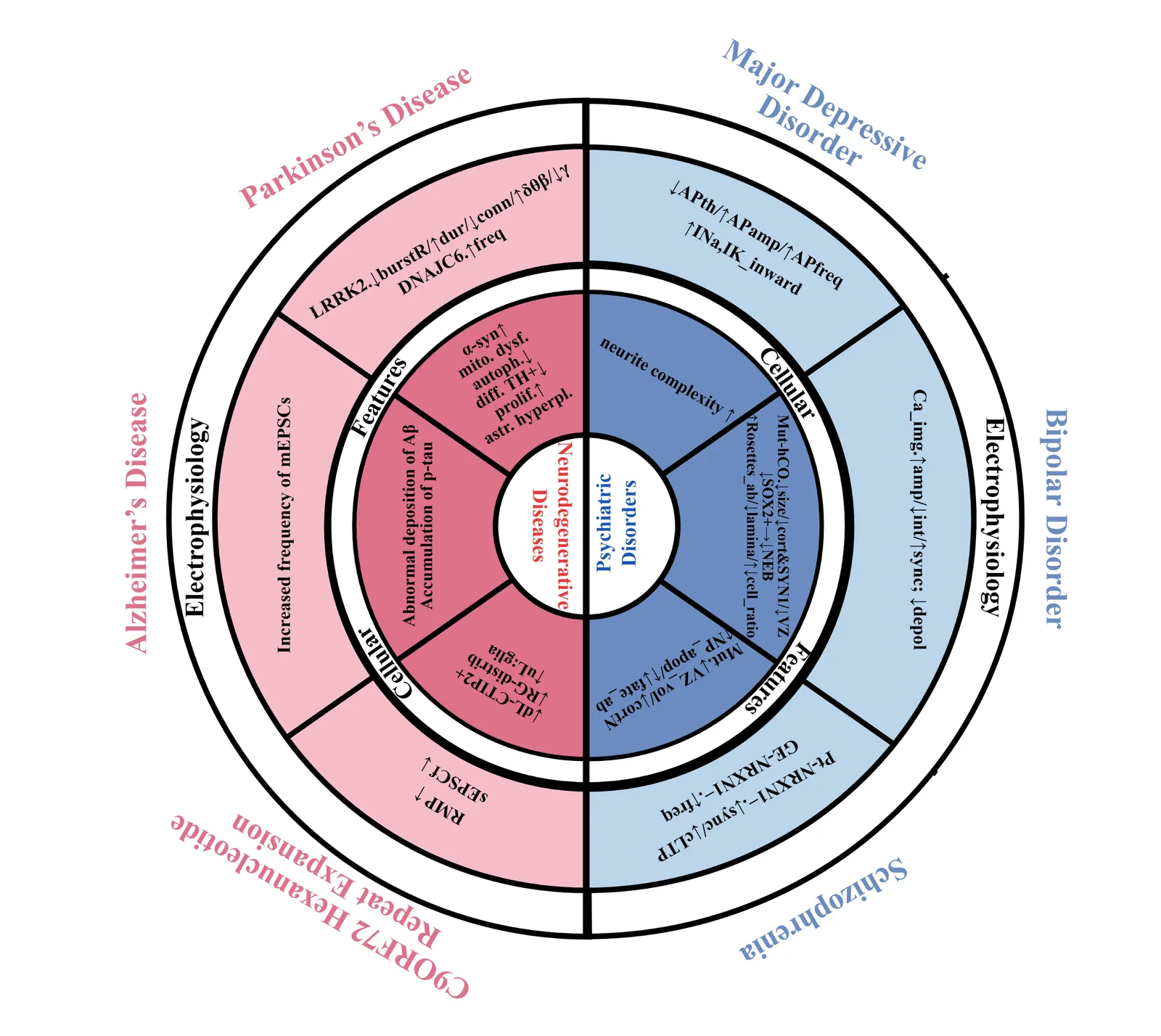
**Cellular and electrophysiological features of hCO models of psychiatric disorders and neurodegenerative diseases (By Figdraw**. ID: WTPSWedd7f)

### 2.4 Epilepsy

The pathological essence of epilepsy stems from disrupted neural network E/I balance, characterized by abnormal local neuronal hypersynchronized discharges and oscillatory activity. This persistent electro-physiological dysfunction leads to progressive structural and functional impairments in neural networks [68]. Characteristic histopathological alterations include selective loss of excitatory/inhibitory neurons in specific brain regions, aberrant axonal sprouting, synaptic remodeling, and glial functional reorganization [68]. Genetic studies have identified over 30 pathogenic genes in autosomal dominant monogenic epilepsy pedigrees, encompassing ion channels, neurotransmitter receptors, transcription factors, and metabolic enzymes. However, most cases exhibit polygenic interactions [68]. Notably, primary epilepsy-associated genetic abnormalities frequently overlap with neurodevelopmental disorders (e.g., Rett syndrome, WOREE syndrome, *UBA5*-related encephalopathy), where mutations disrupt neuronal morphogenesis and functional maturation, inducing persistent electrophysiological abnormalities [29-31, 37, 38, 45]. In model development, Santos et al. [69] induced energy metabolism dysfunction in hCOs via oxygen-glucose deprivation (OGD), resulting in impaired ion channel function, dysregulated neurotransmitter synthesis/release/uptake, neuronal injury/death, and local network disruption, successfully mimicking secondary epilepsy electrophysiological signatures. LFP recordings demonstrate significantly greater power spectral increases in 4-month-old OGD-treated hCOs versus 7-month-old groups, with bumetanide and cannabidiol effectively suppressing these pathological spectral changes [69]. This model provides a critical tool for investigating non-genetic epilepsy mechanisms, though electrophysiological data from secondary epilepsy hCO models remain limited. Importantly, hCOs exhibit inherent limitations in modeling epilepsy pathology: their immature regional architecture and simplified neural circuits constrain translational relevance for studying epileptogenesis. Although hCO-based epilepsy models capture network functional alterations and epileptiform activity, the specific mechanisms linking these phenomena to clinical seizure pathogenesis require further mechanistic validation.

Primary and secondary epilepsy models exhibit distinct mechanistic underpinnings: Genetic epilepsies (e.g., WOREE syndrome, RTT) predominantly manifest ion channel or neurotransmitter pathway dysfunction, driving network hyperexcitability; whereas OGD-induced secondary models display enhanced synchrony due to energy metabolism disruption, reflecting the critical regulatory role of metabolic states on network functionality. Notably, despite both *UBA5*- and *PMM2*-mutant models exhibiting abnormal high-frequency oscillations, the former associates with GABAergic neuron loss while the latter involves glycosylation defects—indicating potential molecular heterogeneity underlying convergent electrophysiological phenotypes. Genetic epilepsy models often overlap with neurodevelopmental syndromes (e.g., Rett, WOREE), making it difficult to distinguish seizure-specific mechanisms from general developmental abnormalities. Electrophysiological findings vary across platforms: patch-clamp and Ca²^+^ imaging typically show hyperexcitability, while network-level recordings (MEA, LFP) reveal partial or age-dependent synchronization. For instance, OGD-induced models exhibit strong low-frequency spectral changes at 4 months but weaker effects at 7 months, highlighting stage-specific vulnerabilities. Recurrent observations—ion channel dysfunction, neurotransmitter imbalance, and network desynchronization—lack standardized validation, leaving unclear whether they reflect universal epileptogenic signatures or context-specific effects of injury, mutation, or culture stress. Furthermore, the immature cytoarchitecture and incomplete excitatory/inhibitory networks in hCOs limit their capacity to model seizure propagation or pharmacoresistant epilepsy. Addressing these gaps will require cross-modal electrophysiology, long-term maturation or assembloid approaches incorporating inhibitory circuits, and comparative studies across monogenic and acquired models, which are essential to reconcile conflicting data and link hCO electrophysiology to clinical seizure dynamics.

### 2.5 Infectious Diseases

#### 2.5.1 Zika Virus

Zika virus (ZIKV), a single-stranded RNA virus of the Flaviviridae family, can cross the placental barrier via mosquito transmission, causing severe congenital central nervous system malformations (e.g., microcephaly) in fetuses [70]. Forebrain organoid infection models demonstrate developmental stage-dependent ZIKV pathogenicity: infection of day 14 hCOs reveals viral tropism for SOX2+ neural progenitors, leading to reduced overall organoid volume and significantly diminished VZ and neuronal layer thickness after 18 days of culture, recapitulating core microcephaly pathology. In contrast, infection of day 80 hCOs shows attenuated viral effects, indicating heightened progenitor susceptibility to ZIKV [12]. Notably, ZIKV infection activates endogenous antiviral signaling and cellular stress response pathways in hCOs [71]. ZIKV organoid models have successfully recapitulated the central mechanisms of microcephaly, exhibiting phenotypes such as reduced organoid volume, thinning of the neural progenitor layer, and diminished VZ thickness, thereby demonstrating developmental damage dependent on the timing of infection. However, current research primarily focuses on morphological and cellular-level alterations, lacking electrophysiological data to support the specific impact of infection on neural network functionality.

#### 2.5.2 SARS-CoV-2

Severe acute respiratory syndrome coronavirus 2 (SARS-CoV-2), known for causing coronavirus disease 2019 (COVID-19) through respiratory system infection, can also breach the blood-brain barrier during early infection, leading to neurological complications [72]. Although current evidence suggests limited productive viral replication in the CNS, peripheral infection disrupts blood-brain barrier integrity via immune-mediated mechanisms and induces neuroimmune homeostasis imbalance, ultimately causing neural dysfunction [73]. Studies using hCO infection models demonstrate that SARS-CoV-2 significantly increases neuronal apoptosis rates, reduces synaptic density, and activates aberrant microglial engulfment of postsynaptic structures (enhanced phagocytic activity by ~3-fold). This synaptic overpruning correlates with persistent interferon responses [74]. Transcriptomic and secretome analyses further reveal upregulated microglial interferon signaling, migration, and synaptic phagocytosis-related genes in infected hCOs, alongside astrocytic activation of interferon-stimulated genes and proliferative signaling, accompanied by endoplasmic reticulum stress, proteostasis imbalance, and autophagy dysregulation [74]. Notably, the viral spike protein induces abnormal neuron-neuron and neuron-glia fusion in hCOs. Calcium imaging shows that ~90% of fused neurons retain synchronized electrical activity with unaltered firing frequencies but exhibit significantly elevated intracellular calcium signal amplitudes. Conversely, ~10% of neurons deeply fused with glia completely lose electrophysiological activity [13]. SARS-CoV-2 models highlight the non-cell-autonomous pathways through which the virus impacts neural function via immune activation and fusion mechanisms, and reveal aberrant microglia-mediated synaptic pruning. However, current analyses of calcium signal changes in fused neurons remain predominantly focused on single-cell events, lacking a systematic functional mapping at the network level. Although synaptic structural alterations have been observed, a direct link to cognitive function or neural circuit dynamics has not yet been established. Future research should prioritize functional assessments and validation of long-term effects.

#### 2.5.3 Herpes Simplex Virus

Herpes simplex virus type 1 (HSV-1) efficiently infects hCOs, with single-cell RNA sequencing (scRNA-seq) analyses revealing significant reductions in oRG populations post-infection [75]. During acute infection, the immediate-early protein ICP4 is widely expressed within hCOs, and infectious viral particles are detectable in culture supernatants. Notably, latency-associated transcripts (LATs) are expressed in a subset of outer-layer cells, indicating HSV-1 can establish latent infection in organoids [76]. Dynamic calcium imaging demonstrates that uninfected hCOs exhibit robust spontaneous calcium activity in TUJ1+ neurons under baseline conditions, with well-coordinated neurite-wide electrophysiological synchronization. However, within 24 hours post-HSV-1 infection, neuronal spontaneous calcium activity is markedly attenuated, disappearing completely by 48 hours, accompanied by reduced neurite counts and diminished residual neurite activity [75]. This rapid, progressive suppression of electrical activity suggests HSV-1 infection directly compromises neuronal structural integrity and functional viability, closely paralleling structural degeneration of neurites and suggesting direct viral cytopathic effects on neuronal integrity as the primary driver of functional decline. It should be noted, however, that electrophysiological data are currently limited to early-stage dynamic changes, with a lack of longitudinal tracking data. While existing evidence primarily supports direct viral impairment of mature neuronal activity, higher-order functional properties—such as network synchrony and synaptic regulation—have yet to be systematically investigated.

#### 2.5.4 Human Cytomegalovirus

Human cytomegalovirus (HCMV) establishes long-term latent infection in hCOs, with viral genomes and IE1 protein expression detectable up to 52 days post-infection, confirming persistent infectivity [77]. HCMV infection induces progressive structural disruption in hCOs, including impaired SOX2+ neural progenitor layer formation, aberrant TUJ1+ neuronal layer development, reduced proliferating cell counts, elevated apoptosis rates, and regional necrotic foci [78]. Additional pathological features encompass vacuolation, cyst formation, and dysregulated expression of neural markers such as β-III-tubulin [77]. Functionally, calcium imaging shows complete loss of electrical activity in HCMV-infected foci (IE1+ regions), while MEA recordings confirm global neural network suppression with significantly reduced spike counts, mean firing rates, and synchronization indices [78]. Critically, treatment with HCMV-neutralizing antibody effectively restores calcium transient frequency/amplitude and prevents network functional impairment [78]. Notably, HCMV induces chronic, spatially localized suppression, where complete electrical silencing occurs in IE1^+^ regions due to necrotic and apoptotic cell loss, whereas surrounding networks remain partially preserved, suggesting that dysfunction reflects regional structural destruction and glial involvement rather than immediate pan-neuronal silencing. Nevertheless, the differential susceptibility of neuronal subtypes, glial cells, and vascular-like structures remains uncharacterized, and the mechanisms underlying functional recovery require further investigation.

Multiple viral infection hCO models reveal distinct mechanisms underlying neurodevelopmental impairment and functional dysregulation across different dimensions: ZIKV, HSV-1, and HCMV consistently demonstrate destruction of the neural progenitor layer (SOX2+) and reduced organoid volume, indicating structural developmental disruption; whereas HSV-1 infection acts acutely, with direct viral cytopathic effects on mature neurons serving as the primary driver of functional decline; in SARS-CoV-2 models, neuronal integration is disrupted indirectly via glial fusion and excessive synaptic pruning, whereas HCMV induces cellular necrosis within IE1+ infected regions, directly abolishing local electrophysiological activity. Notably, the successful reversal of electrophysiological suppression by neutralizing antibodies in HCMV models establishes a functional validation platform for antiviral interventions at the electrophysiological level.

**Figure 4.**
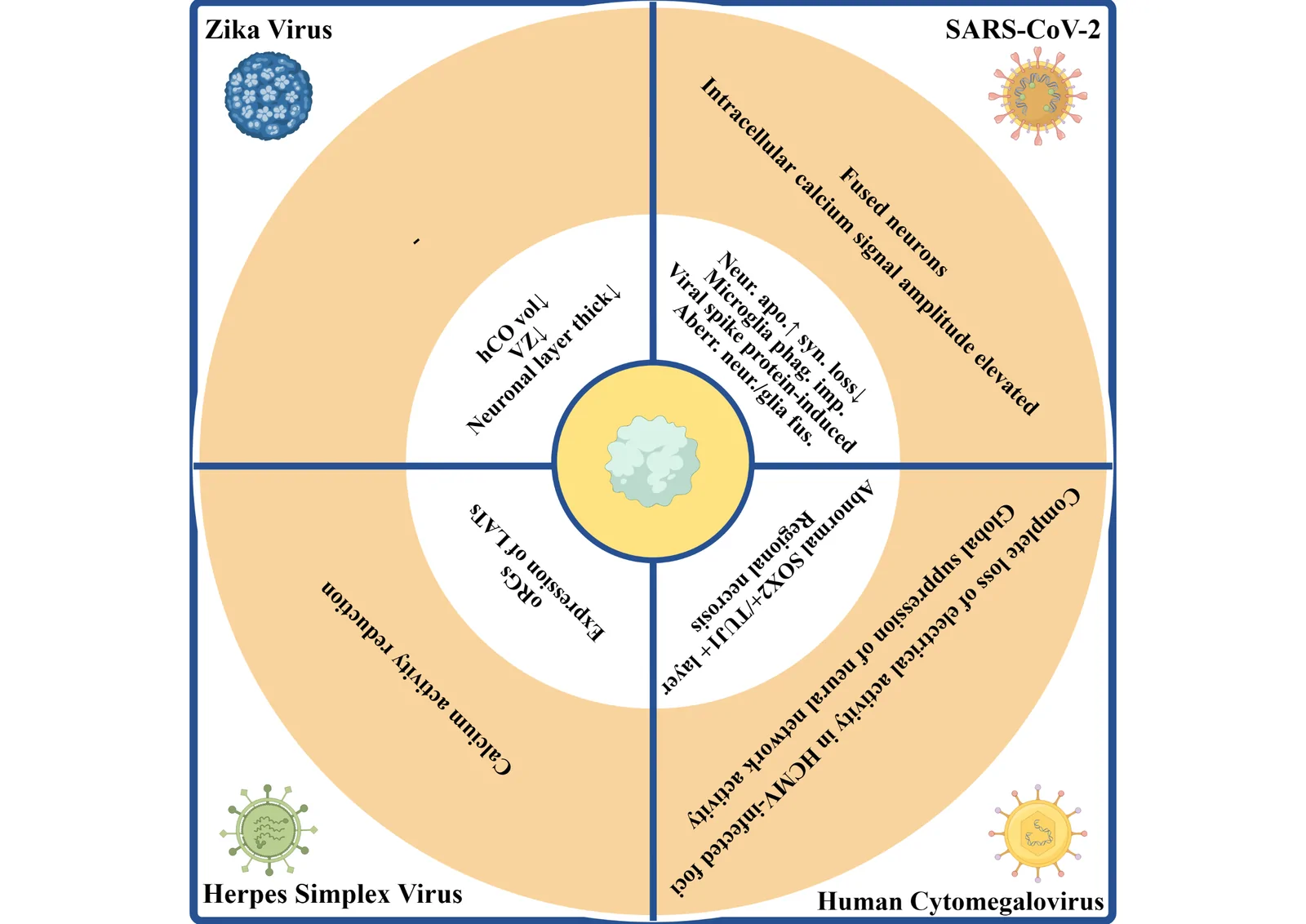
**Cellular and electrophysiological features of hCO models for infectious diseases (By Figdraw**. ID: UTUAP66333)

### 2.6 Traumatic Brain Injury

Traumatic brain injury (TBI), a global public health challenge, has witnessed significant advancements in pathological mechanism research through human hCO models. An in vitro TBI model established via controlled cortical impact (CCI) demonstrates that 220-day-old hCOs post-CCI exhibit astroglial hyperplasia (significant GFAP upregulation), reduced neuronal density (decreased MAP2+ cells), and enhanced neuronal injury markers (NSE accumulation) and apoptotic signaling (cleaved caspase-3 expression). These pathological features closely mirror those observed in in vivo murine TBI models, validating the reliability of hCOs as a TBI research platform [79]. Silvosa et al. [80] elucidated the differential regulatory mechanisms of mechanical stress parameters by simulating blast wave effects on hCO neural networks. Exposing hCOs to 250 kPa and 350 kPa pressure waves (frequency 500-5000 Hz, single 8-ms pulse) revealed distinct outcomes: under 250 kPa pressure, single-neuron firing activity transiently suppressed with increasing frequency, while population spike frequencies and amplitudes synchronously increased post-stimulation, with low-frequency waves (500 Hz) enhancing network oscillatory power and high-frequency waves (5000 Hz) significantly disrupting network synchrony. When pressure increased to 350 kPa, irreversible hyperexcitability in single-neuron firing frequencies emerged, accompanied by further amplification of population spike amplitudes and a 24-hour-delayed enhancement of network oscillatory power. Notably, high-amplitude pressure waves induced diffuse damage in high cellular-density regions of hCOs, identifying amplitude as a critical determinant of structural injury [80].

In therapeutic strategy exploration, hCO transplantation into lesion sites of TBI mouse models exhibits dual effects: on one hand, although surviving graft cells gradually decrease, their neural stem/progenitor cells accelerate differentiation into mature neurons (downregulated SOX2/Ki67 and upregulated TBR1/FOXP2/CTIP2), improving the host microenvironment via secretion of neurotrophic factors (BDNF, NGF, EGF) and synaptic proteins (PSD-95, SYN), while inhibiting glial scar formation (reduced GFAP expression); on the other hand, graft cells extensively migrate along the corpus callosum to ipsilateral/contralateral cortices, thalamus, and hippocampus, achieving structural integration with the host vascular system (co-localization of CD31+ endothelial cells and STEM121+ cells). By day 70 post-transplantation, graft-derived spontaneous electrical activity is detectable in host brains, with behavioral tests confirming significant restoration of spatial learning and memory functions [81, 82]. TBI-hCO models and grafts provide critical support for investigating pathological mechanisms, cell therapy, and neural functional recovery in TBI. However, these findings are predominantly based on specific transplantation time windows and short-term electrophysiological observations, lacking systematic evaluation of long-term graft survival, network integration, and adverse effects (e.g., ectopic projections or scar formation).

A groundbreaking study achieved enhanced neural functional repair through an organoid-brain-computer interface (OBCI) system. Short-term electrical stimulation increased hCO firing rates and high-frequency band LFP power; prolonged intervention further elevated burst activity frequency and duration, enhanced cross-frequency phase-amplitude coupling, and promoted graft-derived neural projections to the cortex, thalamus, and hippocampus. Von Frey tests demonstrated near-normal sensorimotor functional recovery in OBCI-stimulated groups at 180 days, highlighting its potential to repair damaged neural circuits by strengthening structure-function connectivity [14]. Although this technology requires optimization for biosafety and long-term stability, it establishes an innovative research pathway from mechanistic dissection to clinical translation in TBI treatment.

## 3. Conclusion

hCOs have emerged as unique in vitro models demonstrating exceptional value in neurological disease research. By recapitulating complex pathological processes of neurodevelopmental disorders (e.g., microcephaly, autism spectrum disorder), neurodegenerative diseases (e.g., Parkinson's disease, Alzheimer's disease), psychiatric conditions (e.g., schizophrenia, bipolar disorder), epilepsy, and viral infections, hCOs not only faithfully replicate disease-specific structural abnormalities (e.g., neuronal loss, synaptic remodeling, glial activation) but also systematically unveil core mechanisms of neuroelectrophysiological dysfunction, including network desynchronization and E/I imbalance. Integrated multi-omics analyses and multimodal electro-physiological technologies have precisely mapped the heterogeneous regulatory effects of risk genes on neurogenesis, synaptic plasticity, and circuit functionality. In therapeutic exploration, hCOs serve as robust drug screening platforms, successfully validating the rescue effects of ASOs, lithium, and physical interventions (e.g., low-intensity ultrasound) on pathological phenotypes, while advancing innovative therapies such as OBCI.

Although hCO models have demonstrated robust capabilities in recapitulating cellular and functional phenotypes across multiple neurological disorders, current research still faces significant limitations. Firstly, most studies remain at a nascent stage, confined to single patient-derived iPSCs or specific genetic mutation models with limited sample sizes and substantial background heterogeneity, necessitating systematic validation of the reproducibility and generalizability of their findings.

Secondly, despite the significant progress achieved with hCO models in studying various neurological disorders, the vast majority of published work remains concentrated on early developmental windows. This temporal bias imposes several limitations on modeling late-onset or chronic diseases: (i) many clinically relevant end-stage phenotypes—such as late-stage tau pathology and memory circuit degeneration in Alzheimer's disease, Lewy body accumulation and long-term oscillatory changes in Parkinson's disease, progressive neuromuscular junction degeneration in ALS, and age-associated cognitive decline or recurrent mood episodes in psychiatric disorders—require more mature neural circuits and longer disease courses to manifest; (ii) early molecular or electrophysiological alterations observed in hCOs may primarily reflect “susceptibility” or short-term compensatory states rather than the core pathologies driving long-term disease progression; and (iii) temporal heterogeneity across genes and etiologies—for instance, the late-onset impact of LRRK2 mutations versus the earlier phenotypes observed in DNAJC6 or PINK1 models—further complicates cross-study comparisons.

Thirdly, current studies remain predominantly based on iPSCs from individual patients or specific gene-edited cells, resulting in limited sample sizes and homogeneous genetic backgrounds. Beyond these constraints, additional challenges hinder reproducibility and generalizability. Most available iPSCs are derived from European or North American populations, leading to insufficient ethnic diversity and raising concerns about population-specific disease mechanisms. Moreover, hCOs are inherently heterogeneous, with substantial batch-to-batch variability in size, lamination, and cell type composition, even when derived from the same iPSC line. Finally, inter-laboratory discrepancies in medium composition, induction protocols, and maturation time windows further compromise cross-study comparability. Addressing these issues will require standardized operating procedures, international quality-control frameworks, and inclusion of genetically diverse cohorts in future studies.

Furthermore, current hCO models confront technical challenges: prolonged culture durations, high costs, and insufficient standardization limit experimental reproducibility; the absence of functional vascular systems and neuroimmune microenvironments constrains organoid scalability and pathological fidelity; immature laminar architectures hinder simulations of complex neural circuitry; and existing electrophysiological technologies exhibit bottlenecks in throughput, resolution, and network-level analytical capacity. Future research must prioritize optimizing culture protocols, establishing universal standards, and integrating bioelectronic innovations with next-generation electrophysiological tools. As technological iterations and standardization frameworks mature, hCOs are poised to become pivotal bridges connecting basic research to clinical translation, offering novel paradigms for precision diagnosis and treatment of neurological disorders.
